# Methyl Protodioscin Promotes Ferroptosis of Prostate Cancer Cells by Facilitating Dissociation of RB1CC1 from the Detergent-Resistant Membranes and Its Nuclear Translocation

**DOI:** 10.3390/biom16010038

**Published:** 2025-12-25

**Authors:** Ruonan Wang, Chaoyu Hu, Yi Zhao, Shuhan Wu, Shujuan Cao, Leiming Xu, Dengke Yin, Song Tan

**Affiliations:** 1 School of Pharmacy, Anhui University of Chinese Medicine, Hefei 230012, China; 2022205227015@stu.ahtcm.edu.cn (R.W.); 2023205225026@stu.ahtcm.edu.cn (C.H.); 2023205222003@stu.ahtcm.edu.cn (Y.Z.); shenk0716@stu.ahtcm.edu.cn (S.W.); 19565882670@stu.ahtcm.edu.cn (S.C.); 2 Anhui Institutes for Food and Drug Control, Hefei 230051, China; xuleiming1980@163.com; 3 Anhui Provincial Key Laboratory of Chinese Medicinal Formula, Hefei 230012, China

**Keywords:** methyl protodioscin, detergent-resistant membranes, ferroptosis, prostate cancer, RB1CC1, mitochondria

## Abstract

Methyl protodioscin (MPD), a furostanol saponin found in the rhizomes of Dioscorea plants, has been shown to effectively inhibit proliferation of prostate cancer cells in vitro and in vivo. However, the mechanism underlying this inhibitory action remains unclear. To elucidate the mechanism, we used mass spectrometry to analyze protein rearrangements in detergent-resistant membranes (DRMs). Ferroptosis-related factors were identified in cells in vitro and in vivo. MPD induced the expression of acyl-CoA synthetase long chain family member 4 and reduced expression levels of glutathione peroxidase 4 and solute carrier family 7 member 11. Following MPD treatment, RB1-inducible coiled-coil 1 (RB1CC1) dissociated from DRMs and translocated from the cytoplasm to the nucleus. This translocation induced the expression of ferroptosis-related protein coiled-coil-helix-coiled-coil-helix domain containing 3, promoting ferroptosis in prostate cancer cells. As the nuclear translocation of RB1CC1 was promoted by the JNK signaling pathway, SP600125, a JNK inhibitor, prevented the MPD-induced RB1CC1 nuclear translocation. In summary, MPD induced the dissociation of RB1CC1 from DRMs and its subsequent nuclear translocation, contributing to ferroptosis of prostate cancer cells.

## 1. Introduction

Prostate cancer is the second most common malignant tumor in men worldwide, exceeded in incidence only by lung cancer [1]. Although the 5-year survival rate of early-stage prostate cancer is >95%, it plummets to 30–40% once it becomes castration-resistant prostate cancer [2]. Conventional androgen deprivation therapy is highly effective in patients with early-stage prostate cancer; however, it is minimally effective in advanced-stage patients with metastases. This necessitates the identification of novel targets for prostate cancer treatment to improve the survival rate of patients with advanced disease.

Iron, an essential mineral involved in numerous physiological processes, is vital for maintaining homeostasis and overall health [3]. Intracellular iron primarily exists as Fe^2+^, which is unstable and highly reactive. Owing to its capacity to generate hydroxyl radicals through the Fenton reaction, Fe^2+^ directly reacts with polyunsaturated fatty acids in the plasma membrane, generating a large amount of lipid reactive oxygen species (ROS), which disrupts iron metabolism and triggers cell death via ferroptosis [4]. Unlike cells undergoing more conventional apoptosis or necrosis, cells dying by ferroptosis show unique morphological features, including altered mitochondrial shape and increased membrane density [5]. Currently, induction of ferroptosis is used as an alternative therapeutic option in triple-negative breast cancer [6], colorectal cancer [7], gastric cancer [8], ovarian cancer [9], and liver cancer [10], because stimulation of ferroptosis helps overcome the resistance to conventional chemotherapy, targeted therapy, and immunotherapy. Therefore, targeted induction of ferroptosis in prostate cancer is an attractive treatment strategy, as it has a major advantage of avoiding systemic adverse effects.

The detergent-resistant membranes, also known as DRMs, are located in the membranes of subcellular organelles, including the endoplasmic reticulum, Golgi apparatus, and lysosomes, as well as mitochondria and nucleus [11]. DRMs are important signaling platforms in cells, where several signaling molecules are distributed and aggregated. Therefore, DRMs are essential for multiple pathways. DRMs are critical for iron metabolism in various species. In plants, DRMs regulate trafficking of iron-regulated transporter 1 (IRT1) to the plasma membrane and affect the fusion of vesicles that deliver IRT1 to the plasma membrane, as well as metal transport functions of IRT1 [12]. Ferroportin, the only known iron export protein in mammalian cells, is crucial in iron metabolism recycling, and its function depends on DRMs [13]. When DRMs integrity is disrupted, the ability of ferroportin to export iron is compromised, and intracellular iron (Fe^2+^) levels increase [14], which generates ROS through the Fenton reaction and promotes lipid peroxidation. However, the role of DRMs in ferroptosis remains poorly understood.

Methyl protodioscin (MPD), mainly extracted from the dried rhizomes of Dioscoreaceae plants such as Dioscorea punctata, has been shown to have anticancer and cholesterol-lowering effects [15]. Currently, MPD is widely used in the treatment of cervical cancer [16], pancreatic cancer [17], and diabetes [18]. MPD has been shown to directly induce apoptosis in prostate cancer cells by inducing SHP1 phosphorylation, inhibiting the MAPK pathway, decreasing the expression of p-Erk1/2 and p38, and upregulating the pro-apoptotic factors caspase-3 and Bad [19]. We have previously demonstrated that MPD effectively inhibited cholesterol synthesis, thereby disrupting DRMs structure and inhibiting prostate cancer progression in vivo and in vitro [19]. However, how the disruption of DRMs by MPD translates into an anticancer effect remains unknown.

In this study, building on the previous findings that MPD disrupts the structural integrity of DRMs and inhibits the progression of prostate cancer, we explored the specific mechanism underlying the ability of MPD to suppress proliferation of prostate cancer cells. We show that MPD exerted its inhibitory effects on prostate cancer cells by facilitating the transfer of RB1CC1 from the cytoplasm to the nucleus and promoting the expression of the *CHCHD3* gene encoding a downstream ferroptosis-related protein. Collectively, these changes inhibited the activity of cellular mitochondria and ultimately induced ferroptosis.

## 2. Materials and Methods

### 2.1. Cell Culture

Castration-resistant prostate cancer (CRPC) cell line DU145 and murine prostate cancer cell line RM-1 were purchased from the Cell Bank of the Chinese Academy of Sciences (Shanghai, China). DU145 cells were cultured in the minimum essential medium supplemented with 10% fetal bovine serum, 100 U/mL penicillin, and 100 µg/mL streptomycin. RM-1 cells were cultured in the RPMI-1640 medium supplemented with 10% fetal bovine serum, 100 U/mL penicillin, and 100 µg/mL streptomycin.

### 2.2. Reagents

The following reagents were used in the study: MPD (Cat#54522-52-0, Chengdu Herbal Biotechnology, Chengdu, China) with a purity of up to 98%; 3-(4,5)-dimethylthiahiazo(-z-y1)-3,5-di-phenytetrazoliumromide (MTT) assay reagent (M1020, Solarbio, Beijing, China), BCA protein concentration determination kit (P0012, Beyotime, Shanghai, China), protease inhibitor cocktail (Cat#IKM1010, Solarbio, Beijing, China), 7.5% and 10% rapid gelatin preparation kits (EC0022-A, EC0024-A, SparkJade, Jinan, China), ultrasensitive ECL chemiluminescence kit (ED0015-B, SparkJade, China), radioimmunoprecipitation assay lysate (ED0015-B, SparkJade, China); 4% paraformaldehyde (BL539A, Biosharp, Shanghai, China), Matrigel (HY-K6002, MCE, Shanghai, China), 0.1% crystal violet (G1063, Solarbio), Phos-tag acrylamide (AAL-107, Wako, Osaka, Japan), glutathione peroxidase 4 inhibitor RSL3 (HY-100218A, MCE, Shanghai, China), iron chelator deferoxamine (DFO; HY-D0903, MCE, Shanghai, China), and JNK inhibitor SP600125 (HY-12041, MCE, Shanghai, China).

### 2.3. Cell Viability Assay

Cells were seeded into a 96-well plate at a density of 5 × 10^3^ cells per well. After 24 h, cells were treated with MPD at various concentrations for 24 or 48 h. The MTT solution was added to the culture medium for 4 h. Supernatant was carefully aspirated, 150 μL DMSO was added to each well, and the plate was shaken on orbital shaker (100 rpm) for 10–15 min to dissolve formazan crystals and absorbance was measured at 490 nm using a microplate reader (i3x, Molecular Devices, San Jose, CA, USA).

### 2.4. Cell Scratch Damage Repair and Cell Invasion Assays

DU145 cells (1 × 10^5^) were seeded into 6-well plates and cultured to 80–90% confluence. Subsequently, a straight scratch was made across the cell monolayer using a sterile 200 μL pipette tip. The wound area was rinsed with phosphate-buffered saline (PBS) to remove all detached cells. Fresh medium containing varying concentrations of MPD was then added for continued culture in a 37 °C, 5% CO_2_ incubator with saturated humidity. The repair process was monitored by photographing the wound area at 0 h and 48 h. Each experimental group was set up with three biological replicates, and the wound closure rate was statistically analyzed using ImageJ 1.54 to assess the inhibitory effect of MPD on DU145 cell migration.

Matrigel (20 μL) was applied to the upper chamber of the Transwell and pre-incubated at 37 °C for 30 min to form a gel. Cells (1 × 10^5^) were cultured in the Matrigel-coated upper chamber, with complete medium added to the lower chamber for cultivation. Following 48 h of MPD treatment, cells were fixed with methanol at room temperature for 20 min, stained with 0.1% crystal violet for 30 min, and gently washed with PBS three times to remove non-invading cells on the upper surface of the membrane. Images from three randomly selected areas were counted under a microscope.

### 2.5. Separation and Extraction of DRMs

DU145 cells were lysed for 30 min in the lysis buffer (20 mmol/L Tris-HCl, 150 mmol/L NaCl, 1 mmol/L EDTA, 1% Triton X-100, protease inhibitor cocktail; pH 7.4) at 4 °C. The protein-containing supernatant was added to an equal volume of an 80% sucrose solution and placed at the bottom of an ultracentrifuge tube. To form the density gradient, 35% and 5% sucrose solutions were added carefully, and the mixture was centrifuged at 100,000× *g* for 4 h at 4 °C. Each gradient layer (111 μL), from top to bottom, was collected. Before subsequent analysis, protein concentration was determined using the BCA protein concentration determination kit (P0012, Beyotime, Shanghai, China) assay to ensure equal loading for separation and extraction of DRMs or consistent sample input for mass spectrometry. These samples in DRMs were subjected to protein mass spectrometry (Shanghai Bioprofile Co., Shanghai, China).

### 2.6. Immunoblot Analysis

Cells were lysed by adding the radioimmunoprecipitation assay lysis buffer containing protease inhibitor (P1006, Beyotime, Nanjing, China) to extract total proteins and the protein concentration of the supernatant was quantified using the BCA protein concentration determination kit to standardize sample loading. Subsequently, a 7.5% One-Step PAGE Color Gel Speed Preparation Kit was used to prepare the gels, followed by sample loading and electrophoretic separation of the proteins, which were then transferred to polyvinylidene fluoride membranes. Next, the membranes were blocked with 5% skim milk for 2 h at room temperature and then incubated with the primary antibody overnight at 4 °C. Thereafter, the membranes were incubated with the secondary antibody at room temperature and developed using an enhanced chemiluminescence solution and three independent experiments were performed to ensure the reproducibility of the grayscale analysis results. Grayscale analysis was performed using ImageJ 1.54 (NIH, Bethesda, MD, USA). The primary antibodies used for the immunoblotting and immunohistochemistry analyses are listed in Supplementary Table S1.

### 2.7. Immunofluorescence

Cells were inoculated onto a round coverslip (precoated with poly-L-lysine to enhance cell adhesion) and cultured for 24 h, followed by a 48 h treatment culture, fixed with 4% paraformaldehyde at room temperature, permeabilized with 0.1% Triton X-100, and blocked with 5% bovine serum albumin (BSA). Then, the cells were incubated with the primary antibody in the dark, followed by incubation at room temperature with the secondary antibody, and finally sealed with an anti-fluorescence quenching sealer containing 4′-6-diamidino-2-phenylindole for observation under a fluorescence microscope (DMi 8, Leica, Wetzlar, Germany), with three random visual fields selected per coverslip for imaging. ImageJ 1.54 was used to analyze the position and intensity of the fluorescent signals in the acquired images, and the mean fluorescence intensity of each group was statistically compared to evaluate the expression and localization of the target protein.

### 2.8. Measurements of Glutathione, Malondialdehyde, and ROS Levels

For glutathione (GSH) measurements, DU145 cells were harvested by trypsinization and lysed thoroughly using the lysis buffer provided in the kit. The cell lysate was centrifuged at 10,000× *g* for 10 min at 4 °C to collect the supernatant, which was then used for subsequent detection to avoid interference from cell debris. The absorbance was measured at 412 nm using a SpectraMax i3x microplate reader (Molecular Devices LLC., San Jose, CA, USA). GSH levels in the samples were quantified using a standard curve generated from kit standards.

For the malondialdehyde (MDA) assay, DU145 cells were collected and homogenized with the kit-specific extraction buffer to release intracellular MDA. DU145 cells were processed using the kit according to the manufacturer’s instructions. The absorbance was measured at 532 and 600 nm using a SpectraMax i3x microplate reader (Molecular Devices). MDA levels in the samples were quantified using a standard curve generated from kit standards.

For ROS detection, 2′,7′-dichlorofluorescein diacetate (DCFH2-DA; GC42079, GLPBIO, Montclair, CA, USA), a cell-permeable ROS probe, was used. DU145 cells were treated with MPD at varying concentrations and then collected by centrifugation following trypsin digestion. Prior to probe incubation, cells were washed once with PBS to remove residual medium and MPD, ensuring accurate reflection of intracellular ROS levels. Cells were resuspended in the DCFH2-DA working solution and incubated at room temperature in the dark for 30 min. Following incubation, the samples were centrifuged, and the supernatant was removed. The cells were washed 2–3 times with PBS and resuspended for observation under a fluorescence microscope. ROS levels were quantified using ImageJ 1.54.

### 2.9. Determination of Iron Content and Cholesterol Content

DU145 cells were seeded into 6-well plates and cultured to 70–80% confluence before MPD treatment for 48 h. After treatment, cells were washed twice with cold PBS, harvested by trypsinization, and lysed with the lysis buffer provided in the kit at 4 °C for 30 min with occasional vortexing. Following treatment, iron levels in the treated cells were determined using a cell iron content assay kit (BC5315, Solarbio).

In the animal experiments, tumor tissues were harvested, rinsed with normal saline to remove blood, and homogenized into a fine powder in liquid nitrogen before being mixed with the extraction buffer from the kit. Iron concentrations in tumors from different experimental groups were measured using a tissue iron assay kit (BC4355, Solarbio) after tumor tissue was harvested according to the manufacturer’s instructions.

The content of cholesterol in total protein, non-DRM fractions and DRM fractions were measured using a cholesterol assay kit (A111-1-1, Jiancheng, Nanjing, China).

### 2.10. RNA Extraction and Real-Time Quantitative Reverse Transcription (RT) PCR

Total RNA was extracted from DU145 cells that underwent different treatments using the Total RNA Extraction Kit (R1200, Solarbio) according to the manufacturer’s instructions. Reverse transcription was performed using the Reverse Transcription Kit (BL1013A, Biosharp). RT-qPCR was performed using SsoFast EvaGreen Supermix (Bio-Rad, Hercules, CA, USA) according to the manufacturer’s protocol. The sequences of primers used for RT-qPCR are listed in Supplementary Table S2. The melting curve analysis was used to confirm the specificity of the amplified products.

### 2.11. In Vivo Experiments

Male C57BL/6 mice (4–6-week-old) were selected for in vivo experiments to investigate the specific mechanism whereby MPD inhibits tumor growth. RM-1 cells were cultured to logarithmic growth phase, harvested, and resuspended in sterile PBS at 1 × 10^7^ cells/mL. A total volume of 100 μL of the cell suspension (containing 1 × 10^6^ cells) were injected subcutaneously into the right armpit of C57BL/6 mice and allowed to form tumors. The mice were randomly divided into the following groups: control (saline), low MPD (0.5 mg/kg), and high MPD (1 mg/kg). Mice were injected in the tail vein with saline or MPD dissolved in saline once daily for 15 days. Tumor length and width were measured every 2 days to record tumor growth. Mice were euthanized following the completion of the treatment protocol. All animal protocols were approved by the Committee for the Management and Use of Laboratory Animals of the Anhui University of Traditional Chinese Medicine (Approval No. AHUCM-mouse-2025052). All animals were handled according to institutional guidelines.

The tumor tissues were extracted and divided into samples that were snap frozen for protein extraction and those fixed in formalin, embedded in paraffin, and subsequently sectioned at 5 μm for immunohistochemistry. After dewaxing, the sections were placed in citrate buffer (pH 6.0) for antigen retrieval. Following a wash with PBS, sections were blocked with 5% bovine serum albumin at room temperature. Incubation with the primary antibody was performed overnight at 4 °C, followed by washing with PBS and incubation with the secondary antibody at room temperature. Sections were stained with a 3,3′-diaminodbenzidine solution, counterstained with hematoxylin, and examined under a microscope. The area of positive expression in the acquired images was analyzed using ImageJ 1.54, three random fields of view per slide were selected for quantitative analysis.

### 2.12. Prediction of Palmitoylation Modification Sites

GPSPalm is a large benchmark dataset compiled through literature-based bioinformatics curation and integration with public databases. It contains 3098 unique and non-homologous S-palmitoylation sites across 1618 proteins, all identified through small-scale or large-scale experimental studies [20]. Prior to input, the target protein sequence should be standardized to FASTA format, with irrelevant annotations (e.g., species names) removed to avoid prediction errors. Input the name/ID of the protein sequence for prediction to obtain the positions predicted as S-palmitoylation sites. Each predicted site should be cross-validated by checking its conservation in homologous protein sequences retrieved from the UniProt database to enhance the reliability of the prediction result.

### 2.13. Statistical Analysis

Statistical differences among multiple groups were compared using one-way analysis of variance (ANOVA). Comparisons between the two groups were performed using the unpaired Student’s *t*-test. Differences were considered statistically significant if the null hypothesis could be rejected at the *p* < 0.05 probability level. Data are shown as the mean ± standard deviation (SD) of triplicate experiments.

## 3. Results

### 3.1. MPD Induces Protein Rearrangements in the DRMs

**Figure 1:**
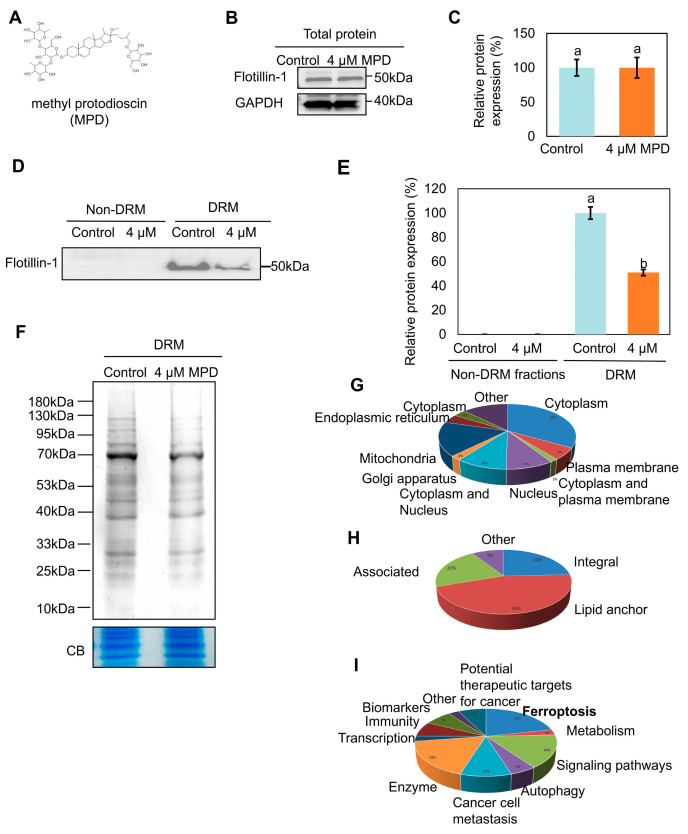
Effect of MPD on DRMs proteins. (**A**) Chemical structure of MPD. (**B**) Immunoblot analysis of flotillin-1 in DU145 cells following MPD treatment. (**C**) Histograms representing the quantification of the flotillin-1 band from (**B**). (**D**) Immunoblot analysis of flotillin-1 in DRMs of DU145 cells following MPD treatment. (**E**) Histograms representing the quantification of the flotillin-1 band from (**D**). (**F**) Coomassie blue (CB) staining of the DRMs fraction of DU145 with MPD treatment. CB of total protein was used as a loading control. (**G**) Subcellular localization of proteins revealed by mass spectrometry in the DRMs fraction of DU145 cells. (**H**) Distribution of membrane proteins revealed by mass spectrometry in the DRMs fraction of DU145 cells. (**I**) Functions of proteins revealed by mass spectrometry in the DRMs fraction of DU145 cells. Data are presented as the mean ± standard error of the mean (n = 3). Statistical significance of differences was analyzed using ANOVA. Significantly different values are indicated by distinct letters (*p* < 0.05). Original images can be found in the Supplementary Materials.

The MPD structure is illustrated in Figure 1A. Following MPD treatment, cholesterol levels in the DRMs fractions were reduced (Figure S2A,B), which is consistent with previous findings [19]. Flotillin-1 (FLOT1), a member of the flotillin family (also designated as the reggie family), is primarily localized to DRMs on the plasma membrane and organellar membranes. Flotillin-1 has been used as a hallmark protein of DRMs [21]. The levels of flotillin-1 in the total protein fractions were not significantly different between the control and MPD-treated groups (Figure 1B,C). However, its levels decreased remarkably in the DRM fractions after MPD treatment (Figure 1D,E), which was consistent with previous findings [19]. Coomassie Blue staining showed that MPD treatment reduced the overall protein content in DRMs (Figure 1F). Mass spectrometry data indicated that DRMs fractions contained protein components associated with different parts of the cell, including the plasma membrane, cytoplasm, mitochondria, and others (Figure 1G, Table S4). Most membrane proteins were lipid anchors (Figure 1H). The functional analysis revealed various biological functions of DRMs proteins, including roles in signal transduction, immunity, and ferroptosis (Figure 1I). Notably, pathway analysis indicated a significant enrichment for proteins involved in ferroptosis regulation, leading us to investigate this form of cell death. These results demonstrate that MPD alters the protein composition of DRMs.

### 3.2. MPD Appears to Promote Ferroptosis in Prostate Cancer Cells

**Figure 2:**
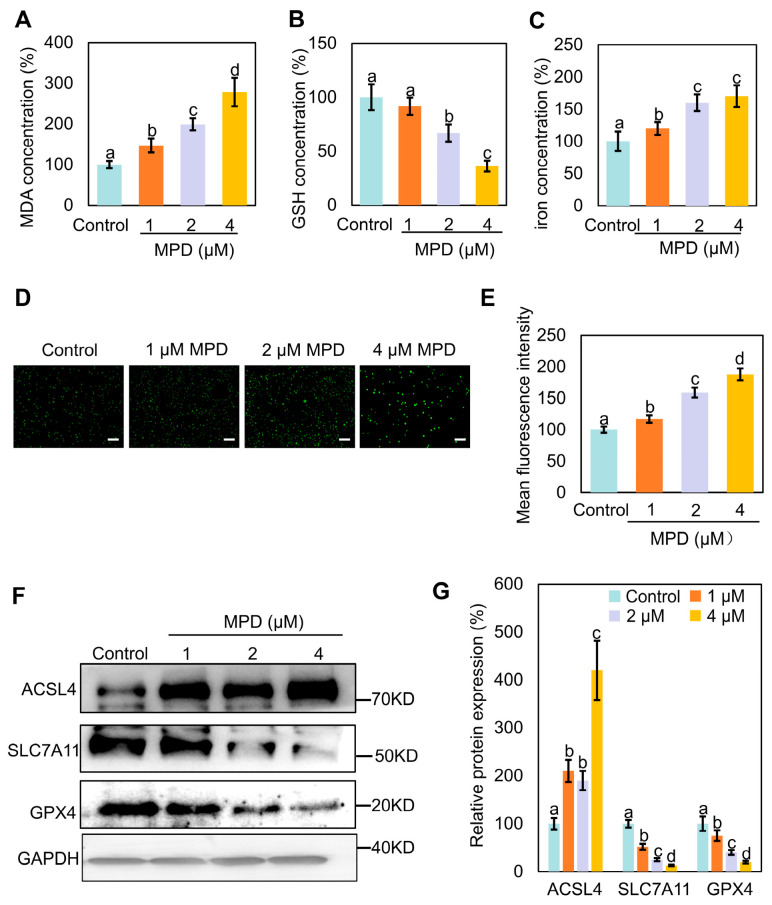
Effect of MPD treatment on ferroptosis in DU145 cells. (**A**) MDA levels in DU145 cells following treatment with MPD at different concentrations. (**B**) GSH levels in DU145 cells following treatment with MPD at different concentrations. (**C**) Fe^2+^ concentrations in DU145 cells following treatment with MPD at different concentrations. (**D**) Assessment of ROS levels using fluorescent probes in DU145 cells following MPD treatment. Scale bar = 100 μm. (**E**) Mean fluorescence intensity analysis of mitochondria from (**D**). (**F**) Immunoblot analysis of ACSL4, GPX4, and SLC7A11 in DU145 cells following MPD treatment. (**G**) Histograms representing the quantifications of the ACSL4, GPX4, and SLC7A11 bands from (**F**). Statistical significance of differences was analyzed using ANOVA. Significantly different values are indicated by distinct letters (*p* < 0.05). Original images can be found in the Supplementary Materials.

MPD may trigger ferroptosis (Figure 1I), so we explored this possibility by measuring MDA, GSH, and iron levels in prostate cancer cells following MPD treatment (Figure 2A–C). MPD significantly increased MDA and iron content, and decreased the GSH content in DU145 cells in a concentration-dependent manner. We used DCFH2-DA as a fluorescent probe and showed that the fluorescence intensity of intracellular ROS was dose-dependently elevated in DU145 cells (Figure 2D,E). These findings confirm that intracellular oxidative stress was increased following MPD treatment.

Next, we examined the expression levels of several ferroptosis-related proteins in MPD-treated prostate cancer DU145 cells using immunoblotting. Acyl-CoA synthetase long chain family member 4 (ACSL4)-catalyzed arachidonic coenzyme A biosynthesis contributes to the induction of ferroptosis by triggering phospholipid peroxidation [22]. Activation of glutathione peroxidase 4 (GPX4) inhibits ferroptosis [23]. Solute carrier family 7 member 11 (SLC7A11) enhances cellular resistance to ferroptosis by maintaining GSH levels and reducing lipid peroxidation, thereby conferring resistance to ferroptosis [24]. Compared with corresponding expression levels in the control group, SLC7A11 and GPX4 expression levels were significantly reduced, whereas that of ACSL4 was significantly elevated following treatment with different concentrations of MPD (Figure 2F,G). In conclusion, MPD treatment increased oxidative stress, decreased cellular GSH levels, decreased GPX4 and SLC7A11 expression, and increased ACSL4 expression, indicating induction of ferroptosis in DU145 prostate cancer cells.

### 3.3. Ferroptosis Inhibitor DFO Reverses MPD-Associated Ferroptosis in DU145 Cells

**Figure 3:**
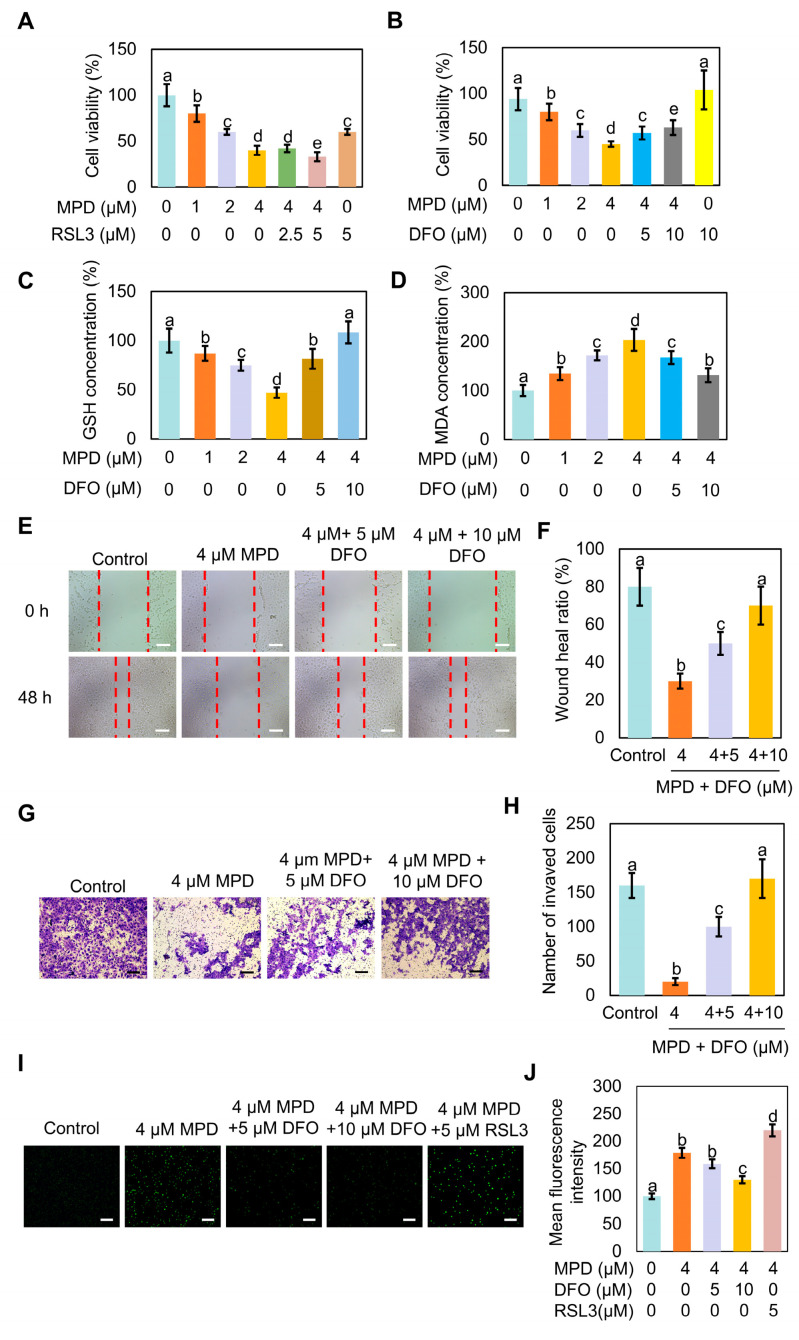
DFO reverses MPD-associated ferroptosis of DU145 cells. (**A**) Viability of human prostate cancer DU145 cells analyzed using the MTT assay following treatment with different concentrations of MPD alone or with ferroptosis activator RSL3 for 48 h. (**B**) Viability of human prostate cancer DU145 cells analyzed using the MTT assay following treatment with different concentrations of MPD alone or with ferroptosis inhibitor DFO for 48 h. (**C**) GSH concentrations in DU145 cells following treatment with different concentrations of MPD alone or with ferroptosis inhibitor DFO for 48 h. (**D**) MDA concentrations in DU145 cells following treatment with different concentrations of MPD alone or with ferroptosis inhibitor DFO for 48 h. (**E**) Migratory activity of DU145 cells following treatment with various concentrations of MPD alone or with DFO for 48 h determined in the cell scratch test. Scale bars = 100 µm. (**F**) Scratch healing ratio of DU145 cells from (**E**). (**G**) Invasive capability of DU145 cells following treatment with various concentrations of MPD alone or with DFO determined in the Transwell assay. Scale bars = 100 µm. (**H**) Numbers of invading cells from (**G**). (**I**) Assessment of ROS levels using fluorescent probes in DU145 cells following MPD treatment alone or in combination with RSL3 or DFO. Scale bar = 100 µm. (**J**) Mean fluorescence intensity analysis of mitochondria from (**I**). Statistical significance of differences was analyzed using ANOVA. Significantly different values are indicated by distinct letters (*p* < 0.05).

To confirm the involvement of ferroptosis in MPD-induced cell death, MPD-treated cells were incubated with the ferroptosis inducer RSL3 or ferroptosis inhibitor DFO. GPX4 serves as a key regulator of oxidative stress-induced cell death, and RSL3 induces ferroptosis by inhibiting GPX4 [25]. DFO is an iron chelator widely employed to reduce iron accumulation and deposition within tissues, thereby inhibiting ferroptosis [26]. The MTT assay showed that MPD effectively and concentration-dependently reduced the viability of prostate cancer cells, and MPD combined with RSL3 had an even stronger suppressive action (Figure 3A). The decrease in cell viability with MPD treatment was reversed by the addition of ferroptosis inhibitor DFO (Figure 3B).

Furthermore, measurements of GSH contents in cells treated with MPD showed that GSH content decreased, whereas that of GSH increased in the co-treatment group, in a DFO concentration-dependent manner (Figure 3C). Measurements of MDA contents in cells treated with MPD showed that MDA content increased, whereas that of MDA decreased in the co-treatment group, in a DFO concentration-dependent manner (Figure 3D)

Cell scratch damage repair experiments showed that the migratory ability of prostate cancer cells was significantly increased in the co-treated group compared with that in the MPD-only group. The recovery of the migratory ability of DU145 cells inhibited by MPD was proportional to DFO concentration (Figure 3E,F). The trans well assay showed that DFO potently reversed the inhibitory effect of MPD on the invasive ability of prostate cancer cells, as significantly more cells crossed the membrane in the co-treated group than in the MPD-only group (Figure 3G,H). The intracellular ROS content was the highest in the MPD + RSL3 co-treatment group and the lowest in the MPD + DFO co-treatment group (Figure 3I,J). Therefore, these results demonstrate that MPD inhibited the migratory and invasive ability of prostate cancer cells by promoting ferroptosis in DU145 cells, and that iron chelator DFO effectively attenuated ferroptosis.

### 3.4. MPD Appears to Induce Ferroptosis in Prostate Cancer In Vivo

**Figure 4:**
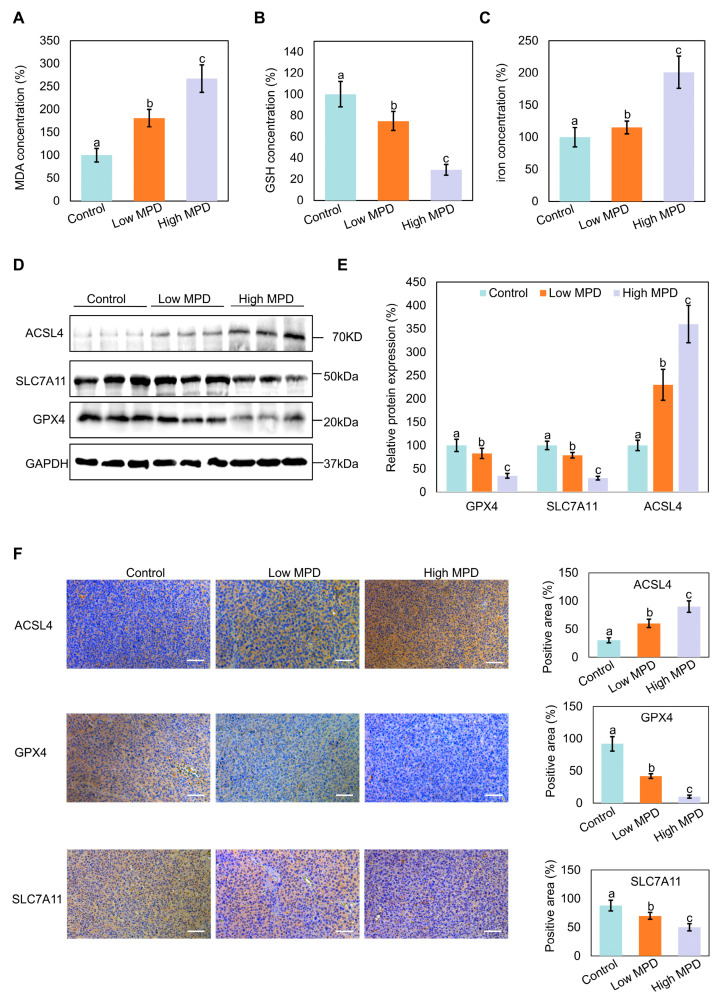
Effect of MPD on ferroptosis in mouse models. (**A**) MDA levels in prostate cancer tumor tissue following treatment with MPD at different concentrations. (**B**) GSH levels in prostate cancer tumor tissue following treatment with MPD at different concentrations. (**C**) Fe concentrations in prostate cancer tumor tissue following treatment with MPD at different concentrations. (**D**) Immunoblot analysis of ACSL4, GPX4, and SLC7A11 in prostate cancer tissue following treatment with MPD. (**E**) Histograms representing the quantifications of the ACSL4, GPX4, and SLC7A11 bands from (**D**). (**F**) Immunohistochemical staining for ACSL4, GPX4, and SLC7A11 expression in prostate cancer tumor tissues. The scale bar is 50 µm. Data are presented as the mean ± standard error of the mean (n = 3). Statistical significance of differences was analyzed using ANOVA. Significantly different values are indicated by distinct letters (*p* < 0.05). Original images can be found in the Supplementary Materials.

To determine the effect of MPD on prostate cancer progression in vivo, the oxidative stress markers MDA, GSH, and iron content were examined in the heterotopic prostate cancer tumors in mice. MPD treatment concentration-dependently increased the oxidative stress levels in tumor tissues compared with that in the control group (Figure 4A,B). The highest tumor iron content was noted in the high-dose MPD group (Figure 4C). The iron levels in the heart, liver, spleen, lung, kidney, and serum of treated mice were not significantly different from the respective levels in the control group (Figure S1). This suggests that MPD only increased the iron content in the tumor, with no effect on the normal tissue.

Immunoblotting and immunohistochemistry showed that MPD treatment decreased the expression of the ferroptosis-related marker proteins GPX4 and SLC7A11 in the tumor tissues of mice with increasing concentration of the treated drug, whereas ACSL4 expression levels increased (Figure 4D–F). These results indicate that MPD appears to inhibit prostate cancer progression in vivo by inducing ferroptosis.

### 3.5. Nuclear Translocation of RB1CC1 Following Its Dissociation from DRMs

**Figure 5:**
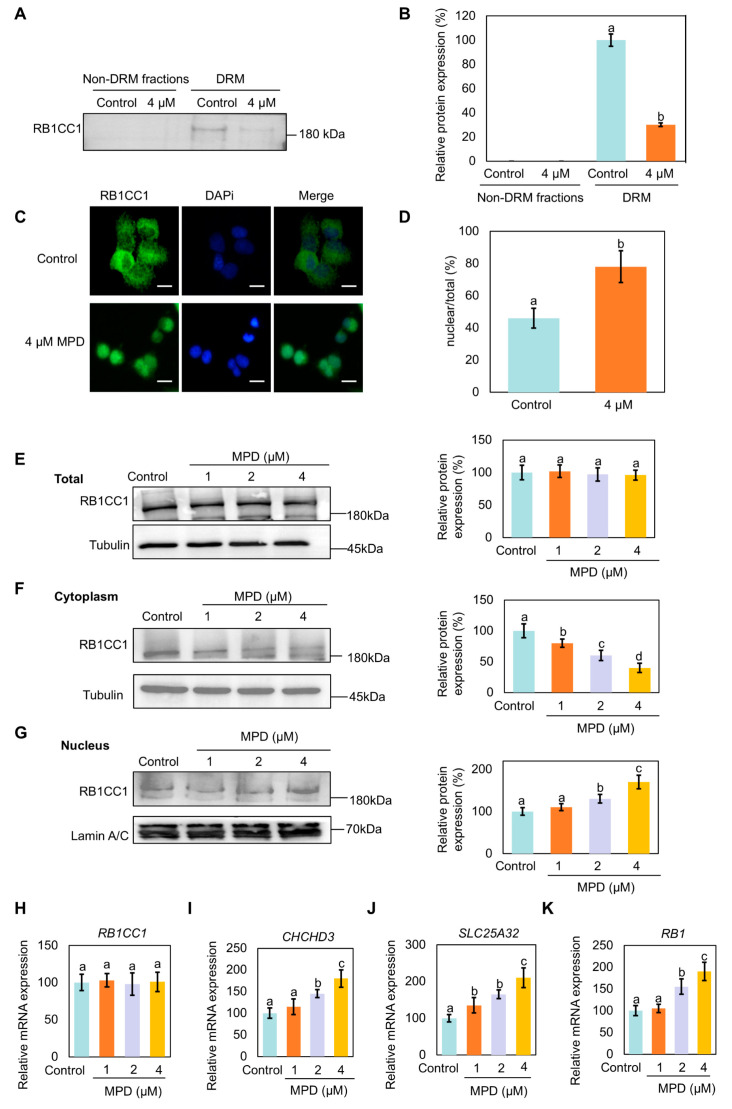
Effect of MPD on the localization of RB1CC1 in DU145 cells. (**A**) Immunoblot analysis of RB1CC1 in lipid DRMs and non-DRMs fractions of DU145 cells following treatment with MPD. (**B**) Histograms representing the quantifications of the RB1CC1 bands from (**A**). (**C**) Immunofluorescence analysis of the intracellular localization of RB1CC1 following treatment with MPD. (**D**) Histograms representing the quantification of the intracellular localization of RB1CC1 from (**C**). (**E**–**G**) Cell fractionation was used to detect the distribution of RB1CC1 following treatment with MPD. (**H**–**K**) Determination of *RB1CC1*, *CHCHD3*, *SLC25A32*, and *RB1* expression levels by RT-qPCR. Statistical significance of differences was analyzed using one-way ANOVA. Significantly different values are indicated by distinct letters (*p* < 0.05). Original images can be found in the Supplementary Materials.

Next, we investigated the mechanism through which MPD appears to promote ferroptosis. A relatively large number of RB1CC1 peptide fragments identified via mass spectrometry suggests a higher abundance of RB1CC1 in the DRMs fraction. RB1CC1 has been shown to be associated with ferroptosis following translocation to the nucleus [27]. RB1CC1 expression in DRMs of the MPD-treated cells was significantly lower than that in DRMs of the control cells (Figure 5A,B). Immunofluorescence experiments showed that the localization of RB1CC1 shifted from the cytoplasm to the nucleus following MPD treatment (Figure 5C,D). To further validate the nuclear translocation of RB1CC1 in the presence of MPD, cytosolic proteins of DU145 cells were plasmid-nucleus separated following MPD treatment at different concentrations. RB1CC1 expression in the cytoplasm gradually decreased and increased in the nucleus with increasing MPD concentration (Figure 5E–G). The findings of the immunoblotting and immunofluorescence experiments suggest MPD promoted the dissociation of RB1CC1 from DRMs and its subsequent translocation from the cytoplasm to the nucleus.

RB1CC1 has been proposed to regulate the transcription of the *CHCHD3* and *SLC25A32* genes by binding to their promoters [27]. The CHCHD3 and SLC25A32 protein is a key regulator of mitochondrial function that indirectly affects ferroptosis by maintaining mitochondrial homeostasis and regulating ROS and iron metabolism [28]. With the increase in the RB1CC1 level in the nucleus, *RB1*, *CHCHD3*, and *SLC25A32* mRNA expression levels also increased in MPD-treated cells (Figure 5H–K). The higher expression of *CHCHD3* increased mitochondrial ROS, ultimately rupturing the mitochondrial membrane and promoting ferroptosis.

Therefore, MPD facilitated the transfer of RB1CC1 from the cytoplasm to the nucleus.

### 3.6. JNK Inhibitor SP600125 Increases MPD-Reduced Cell Proliferation, Migration, and Invasion, and Decreases MPD-Induced RB1CC1 Phosphorylation

**Figure 6:**
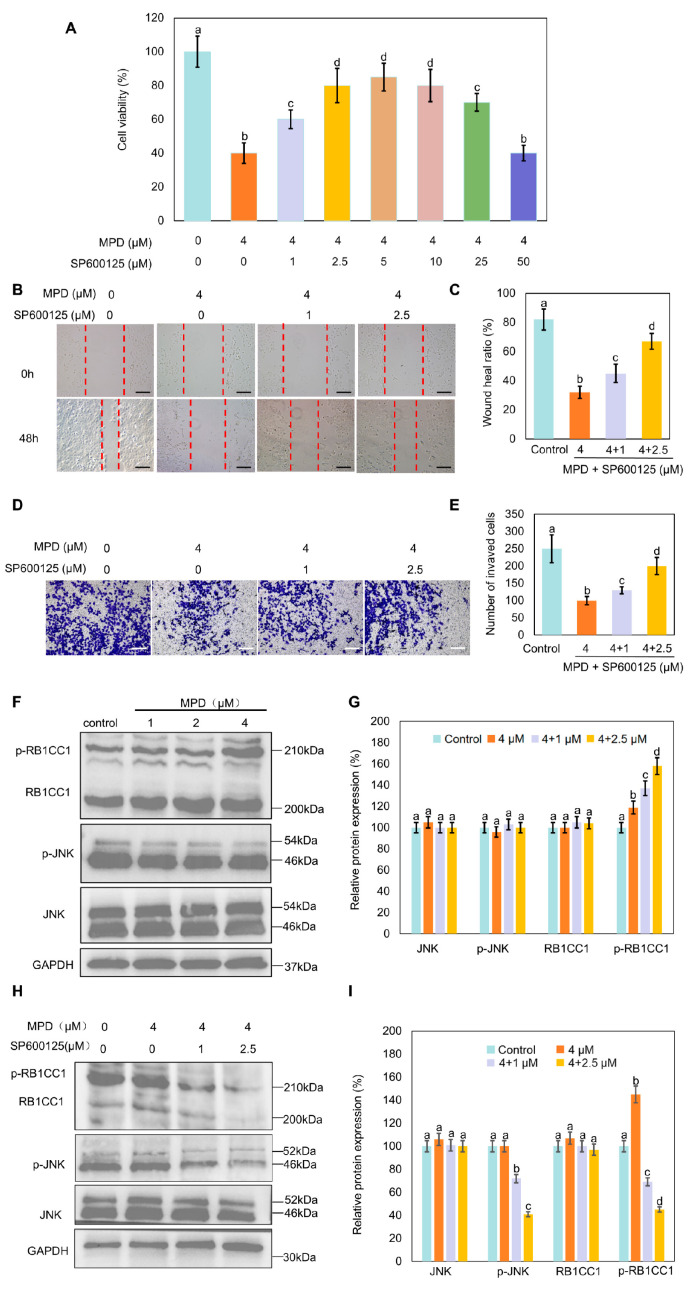
Effect of the JUN signaling pathway on DU145 cell migration, invasiveness, and RB1CC1 phosphorylation. (**A**) Viability of human prostate cancer DU145 cells analyzed using the MTT assay following treatment with different concentrations of MPD alone or with SP600125 for 48 h. (**B**) Migratory activity of DU145 cells determined in the wound healing assay following treatment with various concentrations of MPD alone or with SP600125 for 48 h. Scale bars = 10 µm. (**C**) Wound healing ratio from (**B**). (**D**) Invasive properties of DU145 cells determined in the Transwell assay following treatment with various concentrations of MPD alone or with SP600125. Scale bars = 10 µm. (**E**) Numbers of invading cells from (**D**). (**F**) Immunoblot analysis showing the expression of JNK, p-JNK, and p-RB1CC1 following treatment with MPD at different concentrations for 48 h. (**G**) Histograms representing the quantifications of the JNK, p-JNK, and p-RB1CC1 bands from (**F**). (**H**) JNK, p-JNK, and p-RB1CC1 expression levels analyzed using Western blotting, after 48 h following treatment with MPD alone or in combination with SP600126. (**I**) Histograms representing the quantifications of the JNK, p-JNK, and p-RB1CC1 bands from (**H**). Data are presented as the mean ± standard error of the mean (n = 3). Statistical significance of differences was analyzed using ANOVA. Significantly different values are indicated by distinct letters (*p* < 0.05). Original images can be found in the Supplementary Materials.

JNK phosphorylation effectively activates RB1CC1 by promoting its phosphorylation and nuclear translocation [27]. The relationship between JNK phosphorylation and MPD-regulated RB1CC1 translocation was explored. MTT assay results showed that the inhibition of DU145 cell viability by MPD was reversed by the treatment with SP600125 (Figure 6A). Cell scratch and Transwell assays showed that the inhibition of DU145 cell migratory and invasive abilities by MPD was also reversed by the treatment with SP600125 (Figure 6B–E). Expression levels of p-JNK and p-RB1CC1 were increased in the MPD-treated group compared with those in the control group, whereas the treatment with SP600125 decreased them (Figure 6F–I). These results suggest that MPD induced RB1CC1 phosphorylation and inhibited cell proliferation, migration, and invasion of prostate cancer cells. These effects were dependent on JNK activation.

### 3.7. JNK Inhibitor SP600125 Inhibits MPD-Induced RB1CC1 Nuclear Translocation and Ferroptosis

**Figure 7:**
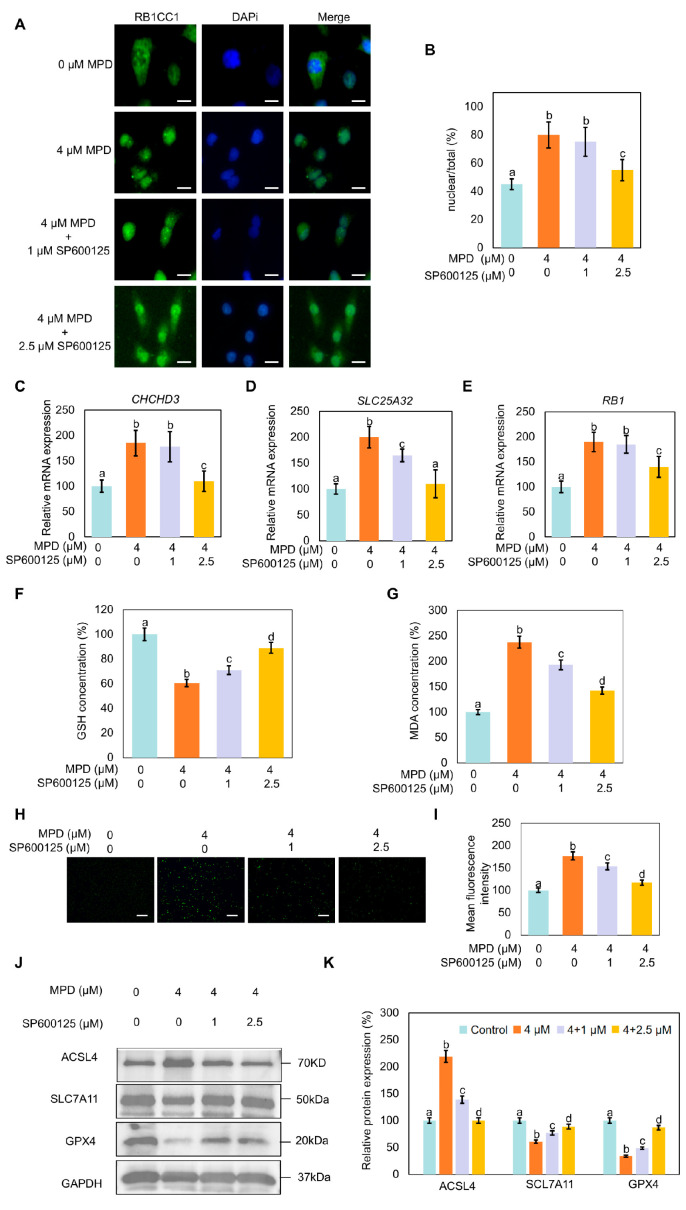
Effect of JNK signaling pathway modulation on RB1CC1 localization and ferroptosis in DU145 cells. (**A**) Immunofluorescence analysis of the intracellular localization of RB1CC1 following treatment with MPD alone or in combination with SP600125. (**B**) Histograms representing the quantification of the intracellular localization of RB1CC1 from (**A**). (**C**–**E**) Determination of *CHCHD3*, *SLC25A32*, and *RB1* expression levels by RT-qPCR. (**F**–**I**) GSH, MDA, and ROS levels in DU145 cells following treatment with MPD alone or in combination with the SP600125. (**J**) Immunoblot analysis of ACSL4, GPX4, and SLC7A11 expression levels in DU145 cells following treatment with MPD alone or in combination with SP600125. (**K**) Histograms representing the quantifications of the ACSL4, GPX4, and SLC7A11 bands from (**J**). Data are presented as the mean ± standard error of the mean (n = 3). Statistical significance of differences was analyzed using ANOVA. Significantly different values are indicated by distinct letters (*p* < 0.05). Original images can be found in the Supplementary Materials.

To further investigate the role of JNK in the MPD effects on RB1CC1 nuclear translocation and ferroptosis, RB1CC1 cellular localization following MPD treatment alone or in combination with SP600125 was examined. Treatment with MPD caused the translocation of RB1CC1 to the nucleus; however, this was reversed following treatment with SP600125 (Figure 7A,B). Furthermore, SP600125 reduced the MPD-induced expression *CHCHD3*, *SLC25A32,* and *RB1*, which encode proteins downstream of RB1CC1 (Figure 7C–E).

Compared with the effects of MPD-only treatment, the co-treatment with SP600125 increased GSH levels and reduced MDA and MPD-induced ROS levels in cells (Figure 7F–I). Immunoblotting showed that MPD treatment decreased expression levels of GPX4 and SLC7A11, but increased ACSL4 expression. The opposite trends in the modulation of these proteins were noted in the SP600125 co-treatment group compared with the levels in the control group (Figure 7J,K). These results collectively indicate that MPD-induced RB1CC1 nuclear translocation and ferroptosis were dependent on JNK activation.

## 4. Discussion

Ferroptosis is an emerging area in the study of cell death that offers a novel perspective on the pathogenesis of tumors, neurodegenerative diseases, and ischemic injury [29]. Understanding the mechanisms that regulate ferroptosis and the development of specific ferroptosis-targeting interventions have gained traction in clinical research. We have previously demonstrated that MPD, a biologically active compound extracted from the rhizomes of Dioscoreaceae plants, disrupts the DRMs structure by inhibiting cholesterol synthesis and potently suppresses viability of prostate cancer cells in vitro and prostate cancer progression in vivo [19]. Here, we sought to reveal specific mechanisms of MPD inhibitory action on prostate cancer cells. Given that MPD disrupted the integrity of DRMs, we identifed that MPD could suppress prostate cancer cell viability by releasing DRMs-bound proteins, appears to induce ferroptosis. This research provides a connection of DRM integrity with ferroptosis susceptibility in cancer.

The CHCHD3 protein is a key regulator of mitochondrial function that indirectly affects ferroptosis by maintaining mitochondrial homeostasis and regulating ROS and iron metabolism, overpressing CHCHD3 also sensitized to ferroptosis [27]. RB1CC1 has been proposed to regulate the transcription of the *CHCHD3* and *SLC25A32* genes by binding to their promoters, nuclear translocation of RB1CC1 increases cellular susceptibility to ferroptosis [30,31,32]. Therefore, it is preliminarily concluded that RB1CC1 is associated with ferroptosis, effectively inhibiting cancer cell viability [27]. Our results revealed that RB1CC1, initially bound to DRMs, dissociated from them in the presence of MPD, entered the cytoplasm, and subsequently translocated to the nucleus. The nuclear translocation of RB1CC1 induced by MPD serves a pivotal role in enhancing ferroptosis sensitivity. In addition, the nuclear translocation of RB1CC1 was affected by JNK phosphorylation, as more protein entered the nucleus under the synergistic action of phosphorylated JNK and MPD. These findings are consistent with the notion that MPD inhibits prostate cancer progression by inducing ferroptosis. The present study still has several limitations: there have been no direct experiments to confirm the connection between MPD, RB1CC1 and CHCHD3. More experiments with knockdown/knockout of RB1CC1 and CHCHD3 or rescue experiments in vitro and in vivo could further confirm that RB1CC1 and CHCHD3 is required for MPD-associated ferroptosis. In addition, this study only used a single cell type for in vitro experiments, which cannot demonstrate that the results have sufficient universality. Future research should conduct further in vivo studies or expand the use of other cell lines. It should be noted that the link between increased Fe^2+^, DRM disruption, and ferroptosis reflects an integrative interpretation based on complementary observations rather than a demonstrated causal sequence and ferroptosis rescue by specific inhibitors (ferrostatin-1, liproxstatin-1, DFO) is required in following research.

Clarification of the specific mechanism of the MPD effect, namely the release of RB1CC1 from DRMs that promoted ferroptosis in prostate cancer cells, prompted another question: how does RB1CC1 bind to DRMs? There are two primary ways in which proteins could bind to DRMs. First, proteins bind to membranes through various specific interactions with membrane lipids and specifically target these proteins to ordered membrane structural domains. For instance, the cholesterol-binding domain of amyloid precursor protein participates in protein–protein interactions but may also be recruited to DRMs structural domains through protein-lipid interactions [33]. Second, proteins can undergo palmitoylation, i.e., binding of saturated palmitic acid to protein cysteines (S-palmitoylation). Hundreds of proteins modified by palmitoylation have been identified to play important roles in signaling, transport, recycling, apoptosis, and protein stability [34]. Palmitoylation differs from other lipid modifications because it is reversible [35], targets many proteins with transmembrane structural domains, and acts as a DRM-binding signal, mediating various actions [36]. Therefore, we utilized the graphic presentation system (GPS) Palm to predict the possible palmitoylation modification sites of RB1CC1 and showed that RB1CC1 likely bound to DRMs through palmitoylation modifications on Cys207, Cys347, Cys379, Cys497, and Cys901. These prediction sites require further comprehensive, definitive experimental validation. Based on the above experimental results, we can observe that MPD effectively disrupted the DRM structure and increased Fe^2+^ content in DU145 prostate cancer cells, consistent with the reported regulation of DRMs by iron. An excessive concentration of Fe^2+^ triggers iron metabolism dysregulation, ultimately leading to ferroptosis.

Ferroptosis is not the only cell death pathway regulated by DRMs. DRMs are crucial in regulating cell death mechanisms, as they affect death receptor aggregation and activation. Many cell death receptors (e.g., Fas/CD95 and TRAIL receptors) and downstream signaling molecules (e.g., FADD and caspase-8) are enriched in DRMs. DRMs provide a platform for these molecules to aggregate, promoting the formation of the death-inducing signaling complex and initiation of apoptotic signaling [37]. DRMs regulate mitochondrial outer membrane permeability and cytochrome c release by affecting the activity and distribution of Bcl-2 family proteins. Some pro-apoptotic proteins (e.g., Bax) translocate to DRMs upon apoptotic stimulation, which promotes the formation of apoptotic vesicles [38,39]. MPD can reduce the core materials required for lipid raft formation by directly interfering with cholesterol synthesis [19]. In here, we identified that MPD appears to induce ferroptosis in prostate cancer. These findings demonstrate that MPD possesses two independent yet synergistic anti-tumor functions. This inherent dual action of MPD provides a new possibility for the treatment of tumors resistant to monotherapy.

DRMs, which are sensitive to oxidative stress, aggregate oxidative stress receptors and effectors as well as regulate ROS production and antioxidant defenses [40]. DRMs also regulate apoptosis, autophagy, and other modes of cell death, and, as a hub of cell membrane function, play a multifaceted role in cancer development and therapeutic resistance. Therapeutic strategies targeting DRMs, including modulation of their cholesterol content, regulation of specific signaling complexes, and development of DRM-targeting drug delivery systems, have unique advantages. Although challenges remain in terms of specificity, delivery efficiency, and clinical translation, DRM-targeted therapies can offer alternatives for overcoming tumor heterogeneity and drug resistance, ultimately improving the outcomes and quality of life for patients with cancer. Future multidisciplinary collaborative research is required to promote the translation of this field into the clinical setting and open new avenues for precision oncology. In this study, we showed that MPD appears to promote the dissociation of RB1CC1 from DRMs and its subsequent nuclear translocation, leading to ferroptosis in prostate cancer.

## 5. Conclusions

In summary, MPD, a furostanol saponin found in the rhizomes of Dioscoreaceae plants, suppressed viability of prostate cancer cells by disrupting the binding between RB1CC1 and DRMs and promoting translocation of RB1CC1 from the cytoplasm to the nucleus. The nuclear translocation of RB1CC1 led to the abnormal expression of downstream proteins associated with ferroptosis, which inhibited mitochondrial function and induced ferroptosis. MPD suppressed the proliferation, migration, and invasive capabilities of prostate cancer cells while promoting their ferroptosis. Therefore, MPD may serve as a natural anticancer agent for prostate tumors.

## Data Availability

The original contributions presented in this study are included in the article/Supplementary Materials. Further inquiries can be directed to the corresponding authors.

## Supplementary Materials

The following supporting information can be downloaded at: https://www.mdpi.com/article/10.3390/biom16010038/s1, Table S1: List of the primary antibodies used for immunoblotting and immunohistochemistry analyses; Table S2: Primer sequences used for quantitative reverse transcription polymerase chain reaction (RT-qPCR); Table S3: All proteins identified in the DRM fractions of DU145 using mass spectrometry; Table S4: The list of the proteins assigned to each functional category; Figure S1: Iron concentration in different tissues and serum of mice following MPD treatment; Figure S2: The cholesterol content in total protein, DRM fractions and non-DRM fractions of DU145 following MPD treatment. Uncropped original images for all Western blots presented in the main figures are provided in the Supplementary Materials.

## Abbreviations

The following abbreviations are used in this manuscript:

MPD	Methyl Protodioscin
DRMs	Detergent-Resistant Membranes
RB1CC1	RB1-Inducible Coiled-Coil 1
CRPC	Castration-Resistant Prostate Cancer
GSH	Glutathione
GPX4	Glutathione Peroxidase 4
GSSG	Glutathione Disulfide
TFR1	Transferrin Receptor Protein 1
Fpn	Ferroportin
CHCHD3	Coiled-Coil-Helix-Coiled-Coil-Helix Domain Containing 3
ROS	Reactive oxygen species
ACSL4	Acyl-CoA synthetase long chain family member 4
SLC7A11	Solute carrier family 7 member 11
