# Supplementary material for: Methyl Protodioscin Promotes Ferroptosis of Prostate Cancer Cells by Facilitating Dissociation of RB1CC1 from the Detergent-Resistant Membranes and Its Nuclear Translocation
Source: Biomolecules. 2025 Dec 25;16(1):38. doi: 10.3390/biom16010038 (PMC12839162; doi:10.3390/biom16010038)

Three repeats of Fig. 1B

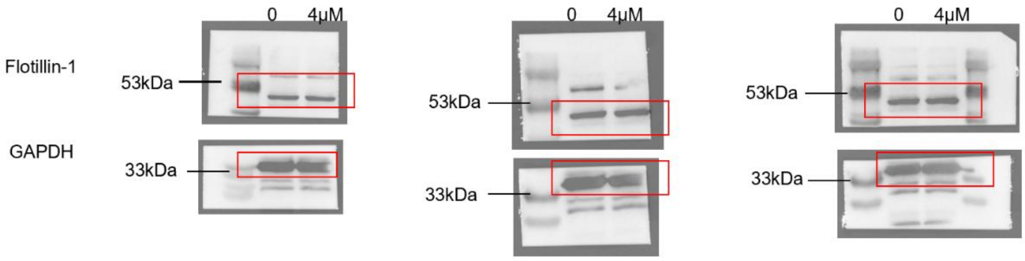

Three repeats of Fig. 1D

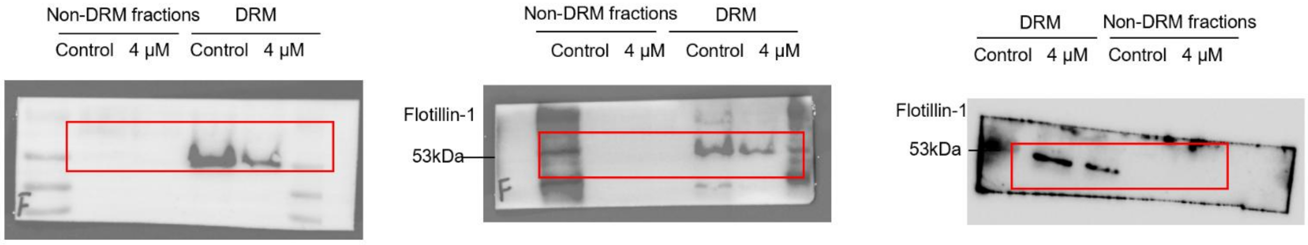

Three repeats of Fig. 2F

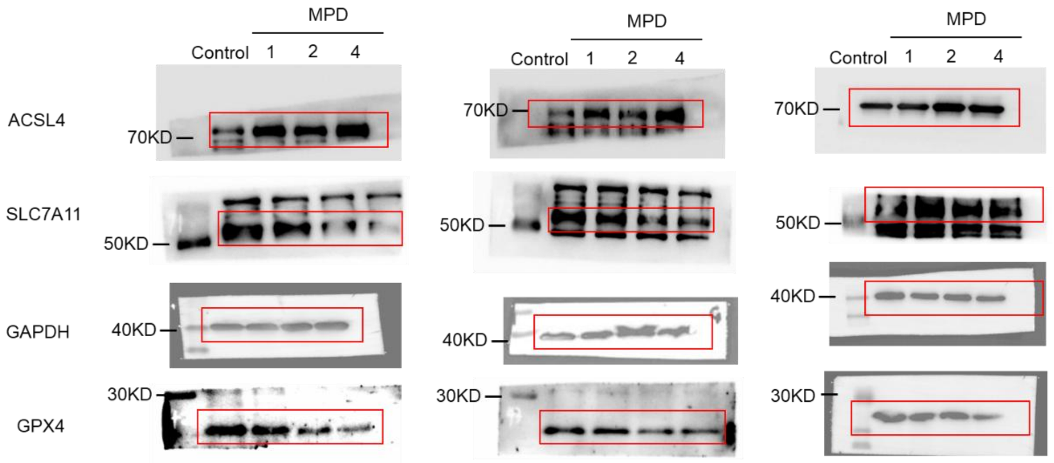

Three repeats of Fig. 4D

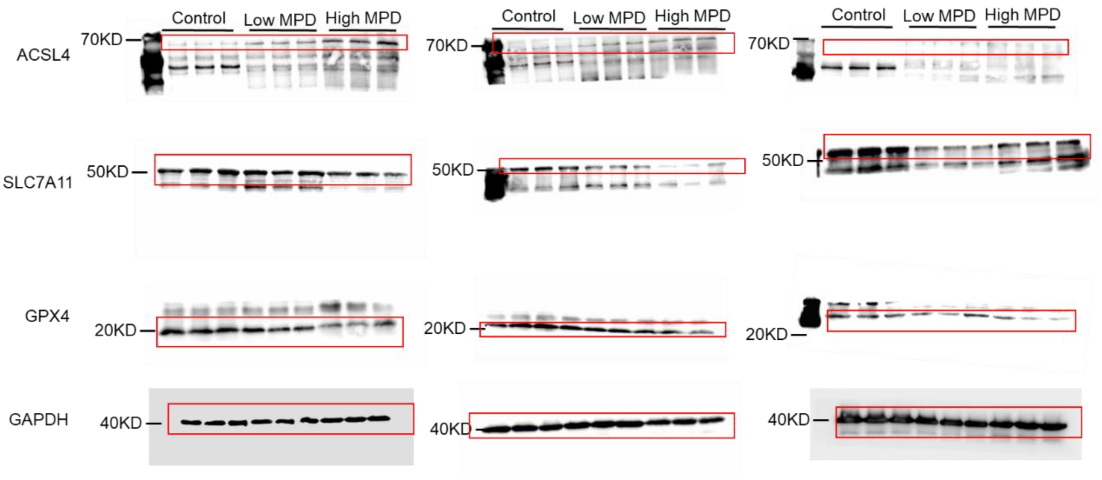

Three repeats of Fig. 5A

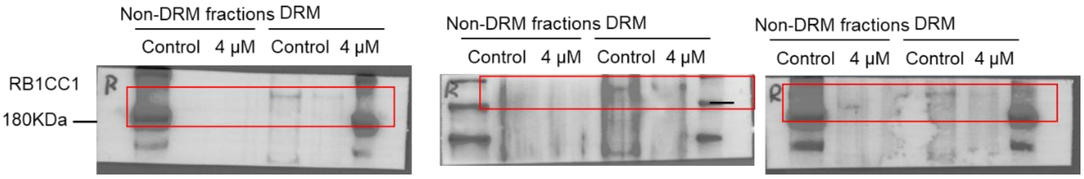

Three repeats of Fig. 5E

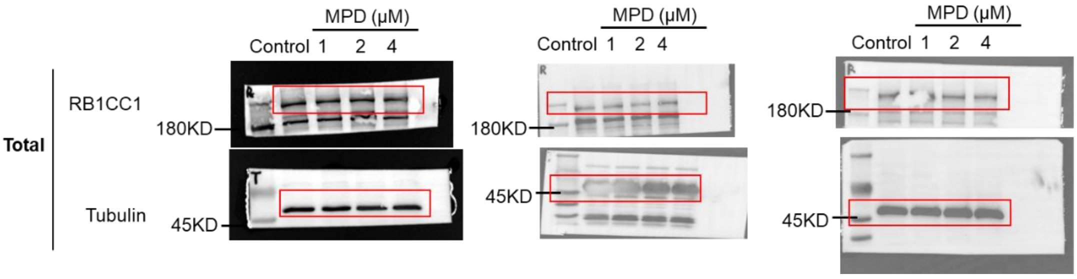

Three repeats of Fig. 5F

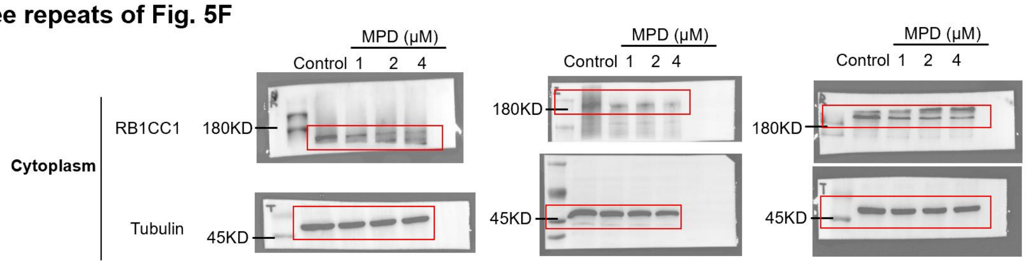

Three repeats of Fig. 5G

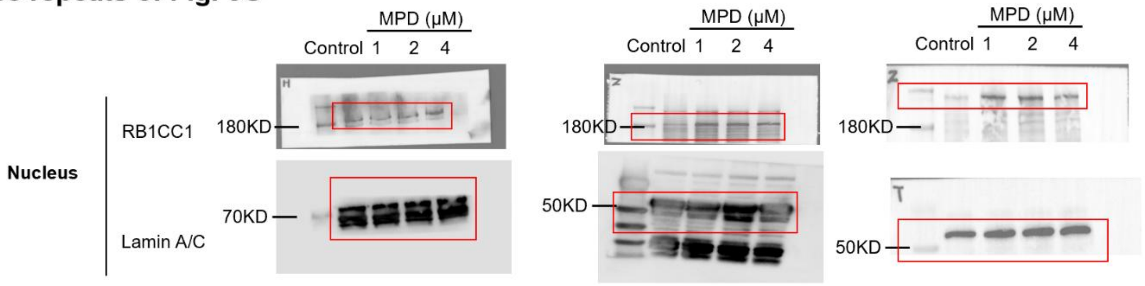

Three repeats of Fig. 6F

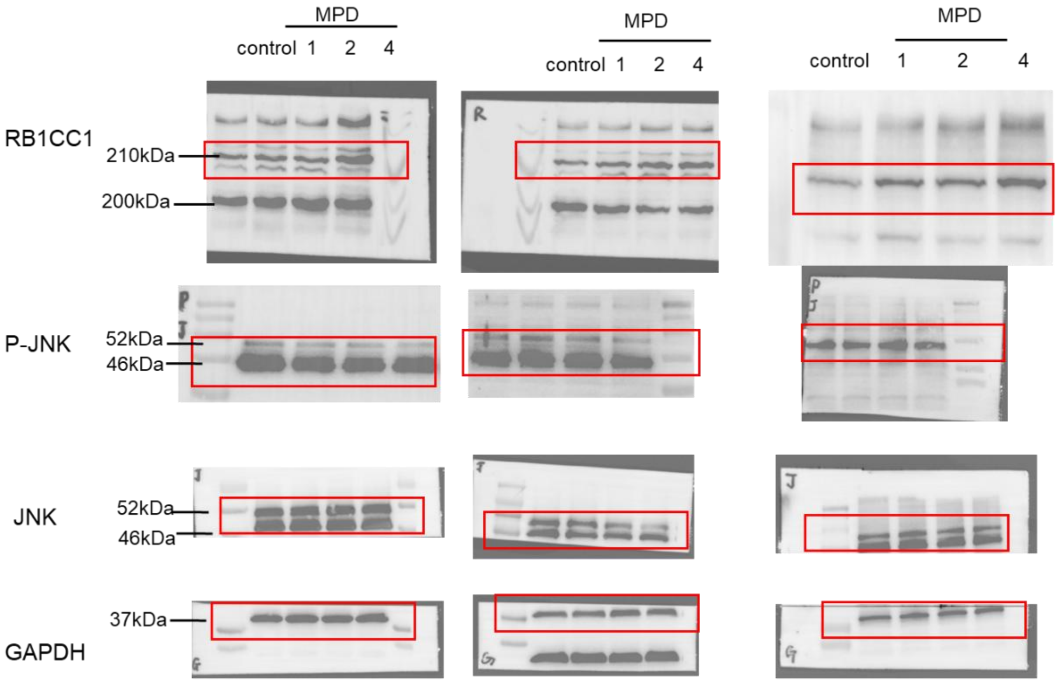

Three repeats of Fig. 6H

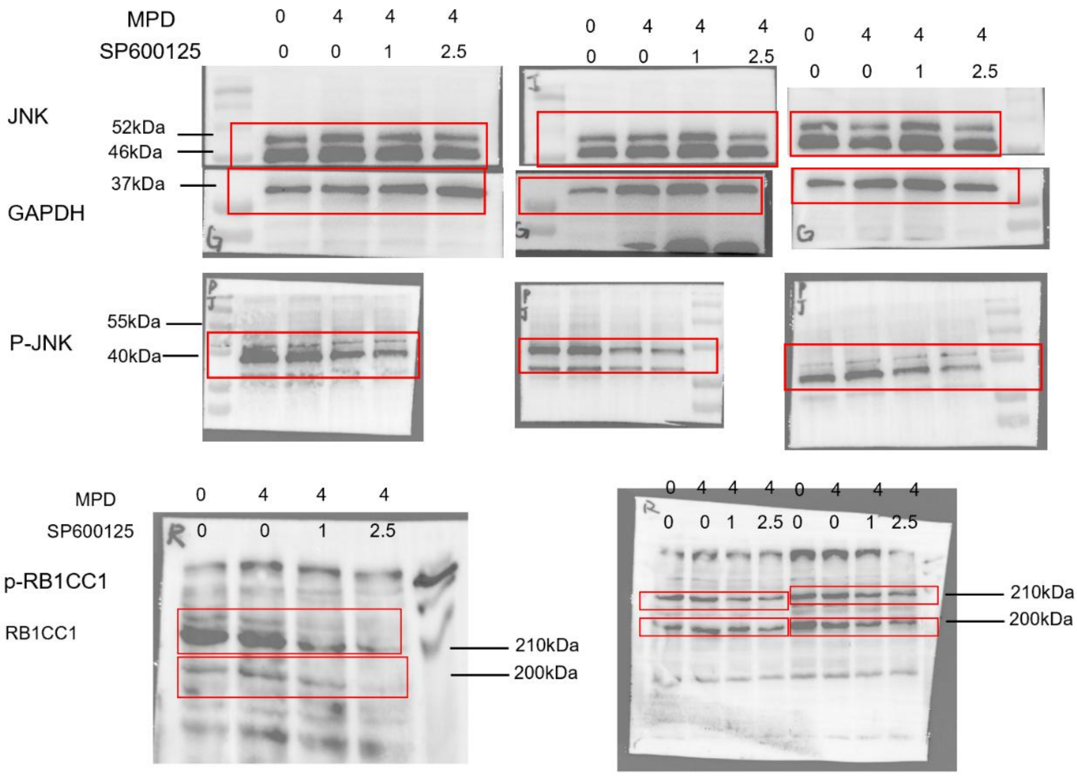

Three repeats of Fig. 7J

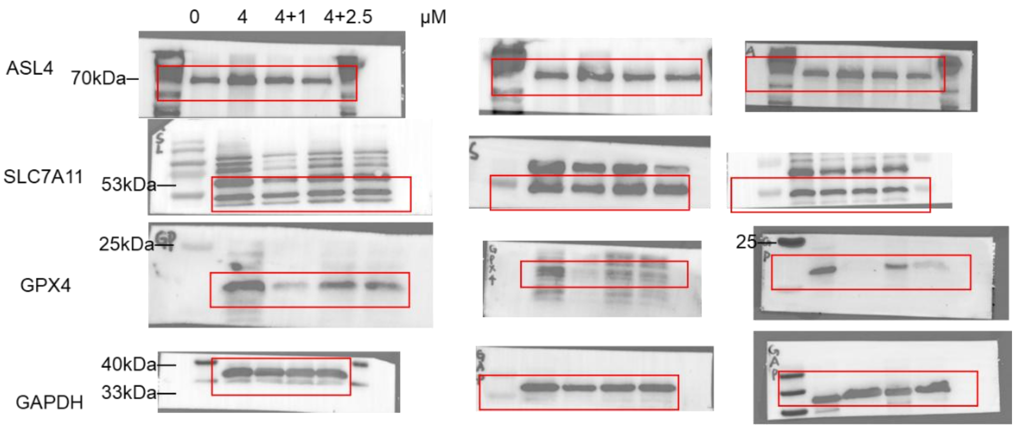

Supplement: Supplementary file 1 [file biomolecules-16-00038-s001.zip › biomolecules-4033168-Supplementary data-Uncropped Immunoblot.pdf]
