# Supplementary material for: Methyl Protodioscin Promotes Ferroptosis of Prostate Cancer Cells by Facilitating Dissociation of RB1CC1 from the Detergent-Resistant Membranes and Its Nuclear Translocation
Source: Biomolecules. 2025 Dec 25;16(1):38. doi: 10.3390/biom16010038 (PMC12839162; doi:10.3390/biom16010038)
Supplement: Supplementary file 1 [file biomolecules-16-00038-s001.zip › Supplementary Figure S1+Figure S2+Table S1+Table S2.pdf]

Methyl protodioscin promotes ferroptosis of prostate cancer cells by facilitating dissociation of RB1CC1 from the detergent-resistant membranes and its nuclear translocation

Ruonan Wang[1, #], Chaoyu Hu[1, #], Yi Zhao[1], Shuhan Wu[1], Shujuan Cao[1], Leiming Xu[2], Dengke Yin[1, 3, *], Song Tan[1, *]

1. School of Pharmacy, Anhui University of Chinese Medicine, Hefei 230012, China

2. Anhui Institutes for food and drug control, Hefei 230051, China

3. Anhui Provincial Key Laboratory of Chinese Medicinal Formula, Hefei 230012, China

#These authors contributed equally to this work

*Corresponding authors: School of Pharmacy, Anhui University of Chinese Medicine, Hefei, Anhui, 230012, China. Email address: tansong_1515@ahtcm.edu.cn (Song Tan, ORCID: 0000-0003-3742-044X); yindengke@ahtcm.edu.cn (Dengke Yin)

Table S1. List of the primary antibodies used for immunoblotting and immunohistochemistry analyses.

Antibody	Cat No.	Company
Anti-RB1CC1	17250-1-AP	Proteintech
Anti-ACSL4	22401-1-AP	Proteintech
Anti-SLC7A11	26864-1-AP	Proteintech
Anti-GPX4	67763-2-Ig	Proteintech
Anti-GAPDH	60004-1-AP	Proteintech
Anti-JNK	R22866	Zenbio

Anti-pJNK	340810	Zenbio
Anti-Lamin A/C	R26947	Zenbio
Anti-Tubulin	AF7011	AFFINITY

Table S2. Primer sequences used for quantitative reverse transcription polymerase chain reaction (RT-qPCR).

Gene name	Forward primer (5′-3′)	Reverse primer (5′-3′)
RB1CC1	GAAAGAGCTTGCTCAGGGATT	TCATCAACTGATTTGCGTGACT
RB1	CTCTGCTCAGGCTTGAGTTTG	GACATCTCATCTAGGTCAACTGC
CHCHD3	GAGGCGGACGAGAATGAGAAC	ACCAGAATACCGCTGAGACTTC
SLC25A32	TCCCCACACCGACAATATAAAGG	CCATGCGATGTTCCAAACAGC
GAPDH	AGACAGCCGCATCTTCTTGT	GATACGGCCAAATCCGTTC

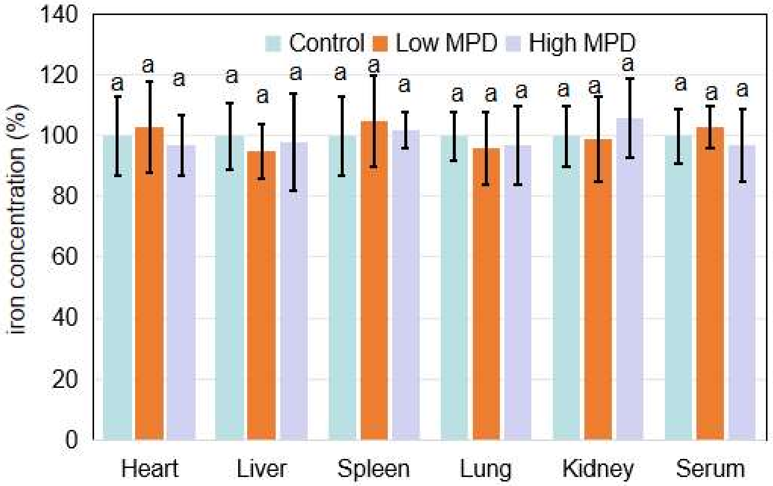

Figure S1. Iron concentration in different tissues and serum of mice following MPD treatment. The iron content in the heart, liver, spleen, lung, kidney and serum of the mice in each group was detected using the kit. Data from three independent experiments (mean standard error n = 3) analyzed using ANOVA presented significant differences indicated by letters ($p < 0.05$).

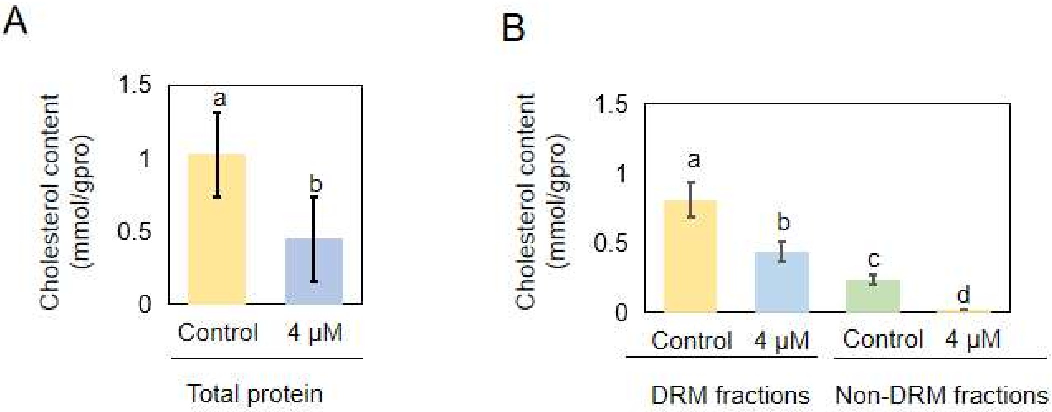

Figure S2. The cholesterol content in total protein, DRM fractions and non-DRM fractions of DU145 following MPD treatment. A. The cholesterol content in total protein of DU145 following MPD treatment. **B.** The cholesterol content in DRM fractions and non-DRM fractions of DU145 following MPD treatment. Data from three independent experiments (mean standard error n = 3) analyzed using ANOVA presented significant differences indicated by letters ($p < 0.05$).
